# CPLLM: Clinical prediction with large language models

**DOI:** 10.1371/journal.pdig.0000680

**Published:** 2024-12-06

**Authors:** Ofir Ben Shoham, Nadav Rappoport

**Affiliations:** Department of Software and Information Systems Engineering, Ben-Gurion University of the Negev, Israel; University of British Columbia, CANADA

## Abstract

We present Clinical Prediction with Large Language Models (CPLLM), a method that involves fine-tuning a pre-trained Large Language Model (LLM) for predicting clinical disease and readmission. We utilized quantization and fine-tuned the LLM using prompts. For diagnostic predictions, we predicted whether patients would be diagnosed with a target disease during their next visit or in the subsequent diagnosis, leveraging their historical medical records. We compared our results to various baselines, including Retain and Med-BERT, the latter of which is the current state-of-the-art model for disease prediction using temporal structured EHR data. In addition, we also evaluated CPLLM’s utility in predicting hospital readmission and compared our method’s performance with benchmark baselines. Our experiments ultimately revealed that our proposed method, CPLLM, surpasses all the tested models in terms of PR-AUC and ROC-AUC metrics, providing state-of-the-art performance as a tool for predicting disease diagnosis and patient hospital readmission without requiring pre-training on medical data. Such a method can be easily implemented and integrated into the clinical workflow to help care providers plan next steps for their patients.

## Author summary

We introduce Clinical Prediction with Large Language Models (CPLLM), a novel method that fine-tunes a pre-trained Large Language Model (LLM) to enhance predictions of clinical diseases and patient readmissions. By leveraging historical medical records, we aimed to predict whether patients will be diagnosed with a specific disease or be readmitted. Our method is compared against the current state-of-the-art model for using structured electronic health record (EHR) data. Our findings demonstrate that CPLLM significantly outperforms state-of-the-art models in both PR-AUC and ROC-AUC metrics. Additionally, our method does not require pre-training on clinical data, making it straightforward to implement with existing LLMs. By integrating CPLLM, healthcare providers can make informed decisions about patient care, ultimately leading to better outcomes. CPLLM can be readily adopted within clinical workflows, assisting care providers in planning appropriate next steps for their patients.

## Introduction

Large Language Models (LLMs) are a type of artificial intelligence (AI) tool shown to be effective in performing a variety of natural language processing tasks [1]. LLMs are trained on large amounts of textual data, which allows them to learn the statistical relationships between words and phrases. LLMs are used for different types of tasks, including natural language comprehension, natural language generation, knowledge-intensive tasks, and reasoning [2]. This makes them well-suited for tasks that require understanding the meaning of a text, such as text classification [3, 4] and even clinical predictions in the medical domain [5, 6].

Clinical predictions are used to estimate a patient’s susceptibility to disease, gauge the likelihood of treatment response, or forecast the course of a given medical condition. [7, 8]. These predictions have been implemented via classical models such as logistic regression [9] and random forest models. However, these traditional methods do not model the order of medical events (diagnoses, procedures, medications, etc.). Instead, they rely solely on the absence or presence of these events (features).

Modern event order prediction models, which are more advanced than the traditional predictive models mentioned above, are based on recurrent neural networks or transformers, of which the latter have been shown to be superior [10], including BERT-style models like BERT [11], RoBERTa [12], and Deberta [13]. GPT-style language models comprise another transformer-based architecture. These GPT models are trained to generate the next token in a sequence. GPT models are used in a wide range of downstream tasks such as summarization, translation, and the answering of questions. [14]. Notable GPT models include LLaMA [15], Falcon [16], Bloom [17], and GPT4 [18].

The significance of the mentioned language models for handling sequential data is particularly important in the context of clinical predictive models that rely on Electronic Health Record (EHR) data. Structured EHR data encompasses a patient’s clinical history, which is notable for its irregular temporal sequence of events and observations [6]. Previous studies have sought to model EHR diagnostic data as a sequence using BERT models including BEHRT [19–22], Med-BERT [23], and Medic-BERT [24] (for predicting length of stay). However, these models represent each diagnosis code as an index and do not address the textual description of the ICD code. In addition, these models are pre-trained using clinical data, and have a limited sequence length input.

There has been limited research focused on developing clinical prediction models using pre-trained LLMs as a starting point for further fine-tuning. One of the main focuses of applying LLMs in the clinic has centered on the chat capability of these models [5, 25] or using an LLM for medical text-based tasks like text generation [26, 27] and text comprehension [28–31]. In addition, [32] proposed a method called ClinTaT for cancer prediction. Their focus was on cancer prognostic prediction using few-shot learning, and their data modeling was not designed for structured EHR data that consists of a sequence of diagnoses. However, we want to harness the power of LLMs to understand sequences of tokens derived from structured EHR data for the specific training of predictive models. For this effort, we chose to present the structured data as a text by representing each medical concept with a word, treating admissions as visits, and considering patient history as a document. The objectives of this study were to develop a novel method for using LLMs to train clinical predictors and to evaluate the performance of this method on real-world datasets.

Our proposed method uses an LLM to predict future patient diagnoses and readmission through the fine-tuning of LLMs. For this approach, medical concepts were represented by text descriptions, and fine-tuning was performed using a prompt that feeds the model with training samples. We used two different LLMs: Llama2, which is a general LLM [15], and BioMedLM, which was trained on biological and clinical text [33]. We used four prediction tasks and two datasets and compared the performance of the resultant models to baseline models.

Our method demonstrates improved performance compared to state-of-the-art methods, even without pre-training on medical data. We have been able to use a general-purpose pre-trained model (Llama2) on non-medical data, effectively adapting it to EHR structured data despite its sequential structure. Our generic method can be used for a variety of tasks and is not specific to any particular LLM. Moreover, our method is also suitable for different clinical domains such as demographics, diagnoses, laboratory test results, measurements, procedures, and more.

**Contributions**.

1. We propose Clinical Prediction with Large Language Models (CPLLM), a novel method for LLM-based clinical prediction that outperforms state-of-the-art models for predicting disease and patient readmission based on structured EHR data. CPLLM does not require pre-training on clinical data and achieves better performance than alternative methods. Moreover, our method has a longer sequence length limit compared to baseline methods.
2. We show that adding additional tokens to the pre-trained tokenizer of the LLM before fine-tuning enhances the clinical predictive model’s performance.
3. Our code is flexible for any LLM, available for use, and can be readily adapted to various clinical prediction tasks.

## Methods

### Disease prediction—Problem definition

Formally, for a given patient *p*, let *n* denote the total number of diagnoses in their medical history. Thus, the patient’s sequence of diagnoses is represented as {*D*_*p*,1_, *D*_*p*,2_, *D*_*p*,3_, …, *D*_*p*,*n*_}, where each *D*_*p*,*i*_ (1 ≤ *i* ≤ *n*) corresponds to a medical diagnosis in the patient’s history. We considered two types of binary diagnostic predictions: next diagnosis and next visit diagnosis.

#### Next diagnosis prediction

Given a patient’s medical history, we predict whether the patient’s next diagnosis will be a target disease of interest. More formally, we predict whether patient *p* will be diagnosed with a specific disease *D*_*x*_ (a text that describes the disease) as the *D*_*p*,*i*+1_ diagnosis, given previous diagnoses. Our model relies on the patient’s medical records up to the *i*-th diagnosis, denoted as {*D*_*p*,1_, *D*_*p*,2_, …, *D*_*p*,*i*_}, where *D*_*p*,*i*_ (1 ≤ *i* < *n*) indicates the most recent diagnosis observed for patient *p*. The predictive model utilizes this patient-specific historical medical information to determine whether patient *p*’s next diagnosis is a specific disease or not.

#### Next visit diagnosis prediction

In some cases we cannot predict the next diagnosis for a patient. Predicting the next diagnosis requires knowledge of the precise timing of each diagnosis. However, these data may occasionally be unavailable, such as when diagnoses are documented at the end of an admission. Therefore, we conceptualize the next visit diagnosis prediction task. Next visit diagnosis prediction is defined as predicting, based on a patient’s medical history, whether the patient will be diagnosed with the disease of interest during their next admission visit. Consequently, in the context of the MIMIC-IV dataset [34], we forecast whether a patient will receive a specific diagnosis in the subsequent admission.

### Prediction of patient hospital readmission

Based on a patient’s medical history, including procedures, diagnoses, and medications, our objective is to forecast whether the patient will experience hospital readmission within the next *X* days. We follow the definition of *X* as specified by the PyHealth benchmark [35]. In our experiments with the MIMIC-IV dataset, we predict hospital readmission within a 15-day window. For the eICU-CRD dataset, the prediction time-frame is 5 days.

### Data

In this study, we used data from the eICU-CRD database [36] and data from the MIMIC-IV database [34]. Our datasets included ICD-9-CM (eICU-CRD) and ICD-10-CM (MIMIC-IV) diagnoses and their descriptions. In the eICU-CRD database, each diagnosis is associated with a timestamp. Consequently, we arranged the diagnoses in chronological order based on their respective diagnosis times. Our disease prediction task aims to anticipate whether the forthcoming diagnosis will correspond to a specific disease. Unlike the eICU-CRD dataset, the MIMIC-IV data lacks information on the exact timing of each diagnosis assignment. However, it provides start times for admission and discharge times for each patient. As a result, our prediction task for this dataset revolves around determining whether a patient will be diagnosed with a specific disease during their subsequent visit.

Med-BERT adopts a pre-training strategy and trains BERT using Masked Language Modeling (MLM) and Length of Stay (LOS) prediction tasks [23]. Therefore, we extracted the necessary data from the databases, including the diagnosis codes for each patient. Additionally, we also included information on the LOS of each admission and the number of visits of each patient. However, in our approach, we did not conduct an additional pre-training step, as we focused on LLM fine-tuning. In our proposed method, it is not required to note during which visit each diagnosis was made. Furthermore, the duration of hospital stay is not required.

#### Data Preprocessing

For the prediction of readmission, we followed PyHealth’s data preprocessing methodology. We included drugs, procedures, and diagnosis codes alongside their respective descriptions. Additionally, we incorporated both ICD-9 and ICD-10 codes and convert them to Clinical Classification Software (CCS) codes [37]. For drugs, we converted the codes to ATC codes [38]. For procedures, we included ICD-9 and ICD-10 procedure codes and converted them to CCS codes using PyHealth. For diagnostic prediction based on the MIMIC-IV dataset, we excluded patients with only one visit, as there was no medical history for such cases. Similarly, for the eICU-CRD dataset, patients with just one diagnosis were removed. We also excluded patients who have the disease we are trying to predict at the first visit (or the first diagnosis for eICU-CRD data). We converted our ICD-10 codes to their corresponding CCS categories for MIMIC-IV, while for eICU-CRD, we retained the ICD-9 codes as they were. This decision was motivated by the higher number of ICD-10 codes compared to ICD-9 codes [39]. Based on the sequence of diagnoses for each patient, we determined whether the patient exhibited a specific diagnosis based on ICD diagnosis codes related to the specific disease according to the relevant CCS category [40]. Table 1 provides an overview of the number of patients, the count of final patients after preprocessing, average diagnoses, and average visits for each disease prediction task.

**Table 1:** Task statistics for the prediction tasks.

Dataset	Task	# of patients	Final # of patients	Disease prevalence (%)	Median # of visits (IQR)	Median # of diagnoses (IQR)
MIMIC-IV	Chronic kidney disease	84,453	26,161	8.157	1 (1–2)	11 (7–19)
MIMIC-IV	Acute and unspecified renal failure	84,453	26,736	19.465	1 (1–2)	11 (7–19)
eICU-CRD	Adult respiratory failure	132,677	56,419	14.549	1 (1–1)	1 (1–2)

Disease prevalence denotes the percentage of cases diagnosed with a specific disease. Visit and diagnosis counts are calculated from the patient’s medical history after preprocessing. IQR—Interquartile range.

#### Clinical outcomes

We evaluated our model’s performance through four prediction tasks: prediction of patient hospital readmission and three diagnostic prediction tasks focused on Chronic Kidney Disease, Acute and Unspecified Renal Failure, and Adult Respiratory Failure. The first two diagnoses were derived from the MIMIC-IV dataset, and the last was derived from the eICU-CRD dataset. The corresponding CCS codes for these diseases were 157 for Acute and Unspecified Renal Failure, 158 for Chronic Kidney Disease, and 131 for Adult Respiratory Failure. For each prediction task, patients with specific disease ICD codes were assigned a positive label, and their diagnosis history encompassed all diagnostic codes recorded until the specific code was indicative of the outcome of interest.

### Baseline methods

We conducted a rigorous performance assessment of the CPLLM against three baseline methods. For diagnosis prediction task, the baseline models included Med-BERT with a classification layer [23], logistic regression [9], and Retain—a disease prediction model featuring double GRUs and attention modules [41]. We compared CPLLM with these baseline methods to gain valuable insights into its performance in clinical prediction tasks. The comparison was conducted using two metrics: the area under the precision-recall curve (PR-AUC) and the area under the receiver operating characteristic curve (ROC-AUC). Disease prediction tasks are typically imbalanced, and ROC-AUC is less suitable for binary classifiers with imbalanced data [42]. Therefore, our main evaluation metric was the PR-AUC, although we also report ROC-AUC for consistency with the baseline methods. When predicting readmission, as mentioned earlier, we compared CPLLM with PyHealth baselines, including the following models: ConCare [43], Retain [41], Deepr [44], and GRASP [45].

### Our proposed method

We propose a method called CPLLM. This method involves fine-tuning a LLM using prompts tailored to medical concept sequences. Through fine-tuning using prompts (inputs for LLM guidance), we direct the LLM to grasp intricate relationships among medical concepts.

We utilized two LLMs: Llama2 (13B parameters) [15] and BioMedLM (also called PubMedGPT, 2.7B parameters) [33]. To enhance time and memory efficiency when fine-tuning these LLMs, we used QLoRA [46] and PEFT [47]. QLoRA is a PEFT approach that decreases the number of parameters requiring fine-tuning and also performs quantization [46]. This combined approach effectively optimized the models’ efficiency, enabling single-GPU fine-tuning for both BioMedLM and Llama2 models.

**Fig 1:**
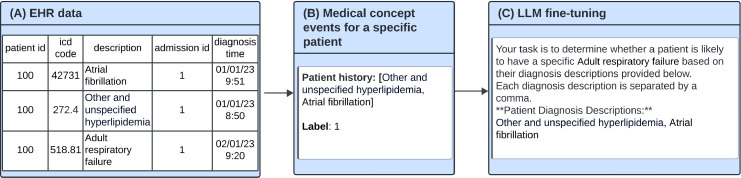
Illustration of the fine-tuning process for diagnostic prediction. A: An example of EHR structured data. The patient has three diagnoses. B: Patient’s historical data is extracted from the EHR and decoded to a textual list of descriptions. C: The decoded textual data is then injected into a designed prompt for fine-tuning the LLM. Fine-tuning prompts consist of a general description, the patient’s diagnostic history, and a label. The label is set to 1 when the patient is diagnosed with the outcome of interest (e.g., Adult respiratory failure in the subsequent diagnosis or during the next admission, depending on the task.

We performed separate fine-tuning of each LLM, leveraging specific prompts tailored to our patients’ medical codes and their corresponding labels. In Fig 1, we present an example of the prompts utilized during the fine-tuning process for both Llama2 and BioMedLM. We also indicated the target disease in the prompt, and the prompts were designed to incorporate patients’ individual medical code histories with the goal of improving the models’ performance. When predicting readmission, the prompt was very similar, but also included drugs and procedures. For diagnostic prediction tasks, we added tokens of diagnosis descriptions missing from the original tokenizer vocabulary of the LLM. We performed an ablation study that compared model performance with and without changing the vocabulary of the pre-trained tokenizer.

For the clinical prediction downstream task, we performed fine-tuning as depicted in Fig 1. Each sample in our training data consisted of a prompt (text) and a label. We used prompts to ask the LLMs to generate a single binary token (0 or 1) in response, by adding a fully connected classification layer as the final layer of the LLM corresponding to the number of labels. We used QLora [46] for our fine-tuning process and froze all layers except the linear layers of the LLM. By training the models with all patient data using Binary Cross Entropy loss for the specified number of epochs, we obtained the fine-tuned LLM tailored to our specific clinical prediction task.

## Experiments

### Experimental setup

When predicting readmission, we compared our method to the PyHealth benchmark. For the diagnostic prediction tasks, we compared our method to three baseline models. The first was a simple logistic regression that does not model the data as a sequence, but as simple independent, unordered variables [48]. For the logistic regression inputs, we used one-hot encoding because it cannot handle text input directly. The second was Retain which is a two-level neural attention model [41]. The third baseline was Med-BERT, the state-of-the-art for structured EHR data for disease prediction. Retain was the baseline for Med-BERT. We split our data using an 70–10-20 ratio to allocate samples to the training, validation, and testing sets, respectively. For Med-BERT, we trained the pre-training model with the MLM and LOS tasks on the training samples from the MIMIC-IV dataset with the TensorFlow package [49]. The training of the Med-BERT’s MLM phase was performed according to the fixed number of steps in the original implementation. The training took about 1.5 days on an RTX1080 GPU. Subsequently, we fine-tuned the pre-trained model for the specific clinical prediction downstream tasks. The Retain and Med-BERT baselines trained for 500 epochs with early stopping based on the PR-AUC values derived from the validation set, using a maximum number of epochs without improvement of 5 [50]. During training of the baselines, we experimented with various batch sizes {32, 100} and different learning rates {1*e*^−5^, 2*e*^−5^}. For each prediction task, we selected the hyper-parameters that achieved the best results on the validation set. For logistic regression training, we utilized the scikit-learn package [51] and trained the model on a CPU. To determine the optimal hyper-parameters for logistic regression, we conducted a grid search encompassing *penalty* (L1 and L2 regularization), *C*, *solver*, and the maximum number of iterations. We explored values of {0.1, 1, 10} for *C*, {‘liblinear’, ‘saga’} for *solver*, and {100, 200, 500} for the number of iterations. We took the best hyper-parameters based on the validation PR-AUC values for each prediction task.

For CPLLM experiments, we fine-tuned two LLMs, Llama2 (13B) and BioMedLM (2.7B), using HuggingFace [52]. [46]. Specifically, we used a learning rate of 2*e*^−5^, Lora alpha of 32, Lora dropout of 0.1, and set the bias to none. Given the resource constraints, we meticulously determined and employed the maximum batch size that our GPU memory could accommodate. We fine-tuned each model over six epochs (four epochs for readmission due to the larger dataset), selecting the best checkpoint based on validation PR-AUC values. Fine-tuning Llama2 for six epochs required about one day of training on an RTX 6000 GPU, while BioMedLM took about two hours on the same hardware. Our fine-tuning process used PEFT, and we did not perform additional pre-training in the clinical domain, yet our CPLLM method outperformed the baseline models.

### Results

#### Diagnostic prediction results

We considered various models for the clinical prediction task: logistic regression, Med-BERT with a classification layer, Retain, and our proposed method, called CPLLM. To examine the statistical significance of the results, we ran each model three times. Table 2 shows the mean and 95% confidence intervals for the PR-AUC and ROC-AUC values derived from these models.

**Table 2:** Performances of various models assessed across multiple tasks and datasets.

Task	Model	PR-AUC	ROC-AUC
Adult respiratory failure	Logistic regression	35.050	74.664
	Retain	34.22 ± 0.299	74.454 ± 0.173
	Med-BERT	34.81 ± 0.208	75.407 ± 0.073
	CPLLM-Llama2	**35.962** ± **0.380**	**76.407** ± **0.262**
	CPLLM-BioMedLM	35.494 ± 0.352	75.975 ± 0.214
Chronic kidney disease	Logistic regression	32.230	83.016
	Retain	31.407 ± 1.379	81.692 ± 0.899
	Med-BERT	33.37 ± 0.891	83.12 ± 0.173
	CPLLM-Llama2	**33.992** ± **1.262**	83.034 ± 0.511
	CPLLM-BioMedLM	33.984 ± 1.077	**83.404** ± **0.429**
Acute and unspecified renal failure	Logistic regression	42.075	77.486
	Retain	43.603 ± 0.409	77.364 ± 0.394
	Med-BERT	42.237 ± 0.408	77.427 ± 0.185
	CPLLM-Llama2	**45.442** ± **0.839**	**78.504** ± **0.684**
	CPLLM-BioMedLM	45.161 ± 1.622	78.484 ± 0.403

The highest score per task is highlighted in bold.

Our findings demonstrate that our method, CPLLM, outperformed all tested models, including Retain, Med-BERT, and logistic regression, across both PR-AUC and ROC-AUC metrics. Specifically, in the context of the Adult Respiratory Failure task, CPLLM-Llama2 achieved a noteworthy PR-AUC value of 35.962%, signifying an absolute improvement of 0.912% over the best-performing baseline model, logistic regression, which obtained a PR-AUC score of 35.05%. This improvement corresponds to a relative enhancement of 2.6% in terms of PR-AUC. Additionally, our method exhibited a relative increase of 5.1% in PR-AUC when compared to Retain and a 3.31% increase when compared to Med-BERT. With respect to ROC-AUC performance, CPLLM also outperformed the baseline models. The Precision-Recall and ROC curves for Adult Respiratory Failure can be found in the supplementary material (see S3 and S4 Figs). Furthermore, CPLLM-Llama2 demonstrated superior performance in this specific task compared to CPLLM-BioMedLM. Logistic regression outperformed Retain in terms of both PR-AUC (35.05%) and ROC-AUC (74.664%), but it also outperformed Med-BERT in PR-AUC, albeit not in ROC-AUC (74.664% vs. 75.407% for Med-BERT).

For Chronic Kidney Disease prediction using the MIMIC-IV dataset, Retain exhibited the worst performance in both metrics. Med-BERT outperformed logistic regression and Retain. CPLLM-Llama2 had the highest PR-AUC score of 33.992%, followed by CPLLM-BioMedLM with 33.984% and Med-BERT with 33.37%. However, in terms of ROC-AUC, CPLLM-BioMedLM outperformed all models with a score of 83.404%, followed by CPLLM-Llama2 with 83.034% and Med-BERT with 83.12%.

For Acute and Unspecified Renal Failure, CPLLM-Llama2 achieved the highest performance metrics, boasting a PR-AUC score of 45.442% and an ROC-AUC score of 78.504%. This signifies a notable improvement of 4.22% in PR-AUC compared with the leading baseline model, Retain, in this task. Additionally, it demonstrated a 1.31% improvement in ROC-AUC compared to the best-performing baseline, logistic regression, with an ROC-AUC score of 77.486%. Furthermore, it is worth highlighting that in this specific task, Retain outperformed Med-BERT in terms of PR-AUC but not ROC-AUC. Additionally, CPLLM-Llama2 demonstrated superior performance compared to CPLLM-BioMedLM. As we found that CPLLM-Llama2 outperformed CPLLM-BioMedLM, the remainder of our analyses will be based on CPLLM-Llama2.

#### Hospital readmission prediction results

To demonstrate the robustness of CPLLM, we expanded our analysis beyond diagnosis to include procedures and drugs. We compared CPLLM against several baseline methods from the PyHealth benchmark. Table 3 presents the results for patient hospital readmission prediction. In the case of MIMIC-IV, CPLLM with LLama2–13B achieved a PR-AUC of 68.986%, outperforming ConCare, the second-best performing model, by 1.46% (absolute). For eICU-CRD, CPLLM exhibited the highest PR-AUC among the baselines, achieving a PR-AUC of 94.115%. Additionally, CPLLM achieved the highest ROC-AUC in both datasets. The Precision-Recall and ROC curves for readmission prediction can be found in the supplementary material (see S1 and S2 Figs).

**Table 3:** PR-AUC and ROC-AUC values for the hospital readmission prediction task for the MIMIC-IV and eICU-CRD datasets.

Dataset	Model	PR-AUC	ROC-AUC
MIMIC-IV	CPLLM-Llama2	**68.986** ± **0.499**	**68.155** ± **0.38**
	ConCare	67.523 ± 0.697	67.242 ± 0.269
	Retain	67.343 ± 0.558	66.893 ± 0.421
	Deepr	66.891 ± 0.604	66.575 ± 0.371
	GRASP	65.656 ± 2.929	65.302 ± 3.369
eICU-CRD	CPLLM-Llama2	**94.115** ± **0.704**	**77.916** ± **1.026**
	ConCare	93.429 ± 0.733	77.024 ± 1.156
	Retain	93.615 ± 0.340	77.149 ± 1.048
	Deepr	93.814 ± 0.422	77.814 ± 0.385
	GRASP	93.677 ± 1.824	77.515 ± 3.899

The highest score per dataset is highlighted in bold.

### Ablation study

We conducted an ablation study to investigate the impact of adding tokens to the pre-trained tokenizer of the LLMs before fine-tuning. Table 4 provides a comprehensive overview of the associated PR-AUC and ROC-AUC values, comparing scenarios with and without the addition of extra tokens. For the task of predicting Acute and Unspecified Renal Failure, adding the tokens yielded enhancements in both PR-AUC and ROC-AUC for CPLLM-Llama2 (0.499% absolute increase in PR-AUC and a 0.554% absolute increase in ROC-AUC). Similarly, CPLLM-BioMedLM showed substantial improvements with a 1.631% absolute increase in PR-AUC, representing a relative enhancement of 3.746%, and a 0.414% absolute increase in ROC-AUC. In contrast, for the prediction of Chronic Kidney Disease, the inclusion of extra tokens did not significantly impact PR-AUC and ROC-AUC values for CPLLM-Llama2. However, CPLLM-BioMedLM demonstrated improvements, specifically an absolute enhancement of 0.686% in ROC-AUC and an increase in PR-AUC from 32.638% to 33.984%. It is worth noting that the PR-AUC of BioMedLM exhibited less stability, as evidenced by a larger confidence interval when no additional tokens are employed (4.358%). Nevertheless, we conducted two additional runs to get a better estimate of the PR-AUC. Subsequently, we observed that the PR-AUC for these five experiments amounted to 33.078%, and the confidence intervals were reduced to 1.773%. When predicting Adult Respiratory Failure, the presence of additional tokens resulted in improved PR-AUC and ROC-AUC for CPLLM-Llama2, whereas it enhanced PR-AUC but did not influence ROC-AUC for CPLLM-BioMedLM. In summary, the findings of this ablation study suggest that, in the majority of cases (9 out of 12 measurements across three prediction tasks), incorporating the added tokens leads to enhanced performance in clinical prediction tasks.

**Table 4:** PR-AUC and ROC-AUC for CPLLM-Llama2 and CPLLM-BioMedLM, across three distinct medical tasks.

Task	Model	Added Tokens	PR-AUC	ROC-AUC
Acute and unspecified renal failure	CPLLM-Llama2	+	**45.442** ± **0.839**	**78.504** ± **0.684**
		-	44.943 ± 1.268	77.95 ± 0.814
	CPLLM-BioMedLM	+	**45.161** ± **1.622**	**78.484** ± **0.403**
		-	43.53 ± 1.101	78.07 ± 0.625
Chronic kidney disease	CPLLM-Llama2	+	33.992 ± 1.262	83.034 ± 0.511
		-	**34.563** ± **1.578**	**83.178** ± **1.02**
	CPLLM-BioMedLM	+	**33.984** ± **1.077**	**83.404** ± **0.429**
		-	32.638 ± 4.358	82.718 ± 1.191
Adult respiratory failure	CPLLM-Llama2	+	**35.962** ± **0.38**	**76.407** ± **0.262**
		-	35.683 ± 0.164	75.776 ± 0.085
	CPLLM-BioMedLM	+	35.494 ± 0.352	**75.975 ± 0.214**
		-	**35.714** ± **0.516**	75.794 ± 0.194

The Added Tokens column indicates whether additional tokens were incorporated into the pre-trained tokenizer. “+” and “-” respectively indicate that additional tokens were or were not added.

## Discussion

Our proposed CPLLM method outperformed the baselines on all four tasks (3 diagnostic predictions and readmission prediction) across two different datasets. We used the MIMIC-IV and eICU-CRD datasets to assess the model’s ability to handle two diagnostic coding systems (ICD9 and ICD10) and two data types (homogeneous data from the same hospital in MIMIC-IV and multi-center data in eICU-CRD). CPLLM was superior to all baselines. CPLLM-Llama2 was the best model overall, and only for the prediction of Chronic Kidney Disease did CPLLM-BioMedLM outperform CPLLM-Llama2, doing so even then only in terms of ROC-AUC. Using CPLLM-Llama2, we achieved relative PR-AUC improvements of 3.309%, 1.864%, and 7.588% over Med-BERT for these three tasks, and corresponding relative ROC-AUC improvements of 1.326% and 1.391% on the Adult Respiratory Failure and Acute and Unspecified Renal Failure prediction tasks. For the prediction of hospital readmission, CPLLM achieved relative improvements of 2.17% compared to ConCare in terms of PR-AUC for the MIMIC-IV dataset. For eICU-CRD-based predictions of readmission, CPLLM showed a relative improvement of 0.31% relative to the second-best result, Deepr.

For the Chronic Kidney Disease task, CPLLM (both CPLLM-Llama2 and CPLLM-BioMedLM) demonstrated superior performance over logistic regression in terms of PR-AUC when considering the 95% confidence intervals. However, in terms of ROC-AUC, the logistic regression performance fell within the confidence intervals of CPLLM. This outcome may be attributable to the limited number of positive cases (8% of the labels, as detailed in Table 1, which can significantly impact ROC-AUC, a metric known to be sensitive to class imbalance [42]. As a result, the ROC-AUC values for Chronic Kidney Disease are higher across all models and closer to one another, potentially explaining why CPLLM does not exhibit a substantial advantage over logistic regression in this metric. ROC-AUC was included to maintain consistency with related studies, such as Med-BERT.

We hypothesize that CPLLM’s superior performance compared to the baselines is due to its larger number of parameters and the substantial amount of training tokens used during pre-training. For instance, CPLLM-Llama2 was pre-trained on 2 trillion tokens and has 13 billion parameters [15]. This reasoning may also explain why CPLLM-Llama2 outperformed CPLLM-BioMedLM in nearly all tasks. The greater parameter count and more extensive training data of CPLLM-Llama2, in comparison to BioMedLM’s 2.7 billion parameters and 34.6 billion tokens, provide a substantial advantage, despite BioMedLM being pre-trained on PubMed abstracts and full articles [33].

In addition, we found that including additional tokens in the LLM’s tokenizer before fine-tuning improved the measurement of the prediction model in most cases. For instance, as Llama2 was not initially pre-trained on clinical data, supplementing it with missing description codes can enhance its understanding of the medical domain.

Regarding the comparison between Med-BERT and Retain, in the original Med-BERT paper, improvements over Retain were demonstrated in terms of ROC-AUC for three disease prediction tasks [23]. We also found that Med-BERT consistently outperformed Retain in all prediction tasks based on ROC-AUC. However, it is worth noting that, as previously mentioned, ROC-AUC may not be an optimal metric for imbalanced datasets [42]. In contrast, when considering PR-AUC, Med-BERT exhibited superior performance compared to Retain in two out of three tasks, although it did not outperform Retain in the prediction of Acute and Unspecified Renal Failure (with PR-AUC values of 43.603% for Retain and 42.237% for Med-BERT), despite achieving a higher ROC-AUC than Retain.

### Strengths and limitations

CPLLM has several advantages compared to existing approaches.

First, Unlike existing approaches that necessitate pre-training with medical concept sequences, our method eliminates the need for additional pre-training tasks. For instance, Med-BERT entails both MLM and LOS prediction tasks using patient sequences of medical concepts. Based on our findings and results, it is evident that LLMs possess the capability to adeptly represent sequential clinical data without the need for specific pre-training based on clinical sequences. Beyond that, our method can be used even without the LOS data corresponding to each patient’s hospitalizations, which is required for Med-BERT pre-training. Sometimes, these data are not available, for example, when there is no hospitalization, but rather data collected among patients who visited a physician in outpatient settings, or when LOS data is not available as in claims data.

Second, the strength of our proposed method lies in its remarkable capacity to handle longer sequences compared to the current state-of-the-art models for structured EHR data. With maximum sequence lengths of 1024 tokens for CPLLM-BioMedLM and 4096 tokens for CPLLM-Llama2, our approach far surpasses the limitations imposed by Med-BERT and BEHRT [19]. Med-BERT is constrained by maximum of 512 tokens, which significantly restricts its ability to handle longer inputs [11]. Without the need for additional training, our method also handles longer sequences compared to Hi-BEHRT, which is specially trained and designed to handle sequences with a maximum of 1220 tokens [20].

Third, during the fine-tuning training of CPLLM, it is not necessary to know which diagnoses were given in which visit but only the diagnoses as a sequence. This differs from Med-BERT, which relies on this information for fine-tuning. Notably, we achieved superior performance even without these specific details.

Fourth, CPLLM demonstrated flexibility for various input types and clinical prediction outcomes beyond disease prediction. This was evident in the readmission prediction experiment, where our approach seamlessly incorporated diagnoses, drugs, and procedures into the sequence with minimal adjustments to the prompt text.

While our method demonstrates promising performance in the utilization of LLMs for clinical prediction tasks, it is important to acknowledge several limitations. We pre-trained Med-BERT on the MIMIC-IV dataset rather than a large corpus as described in the original paper, due to our lack of access to larger datasets and the unavailability of pre-trained Med-BERT weights, which are not publicly accessible because of patient privacy concerns. In addition, while our method accommodates sequences of up to 4096 tokens for CPLLM-Llama2 and 1024 tokens for CPLLM-BioMedLM, our tests did not include exceptionally long sequences that could fully explore the implications of this extended token limit. That is because the datasets we used do not contain very long observations or many diagnoses of a single patient. Moreover, due to the greater number of parameters in LLMs, our method demands more computational resources, inference time, and training time. Specifically, CPLLM-Llama2 had a longer training time than Med-BERT. However, CPLLM-BioMedLM requires less training time compared to Med-BERT. That is because CPLLM-BioMedLM does not require additional pre-training, in contrast with the requirement for MLM and LOS pre-training in Med-BERT. In addition, our method requires using a specific prompt, a requirement that does not apply to the baseline models. As a result, sometimes the prompt must be adapted according to a base model.

### Future work

We hypothesize that combining retrieval augmentation [53, 54] with CPLLM can improve its performance, as it enables inclusion of general updated knowledge about the diseases with which a given patient has been diagnosed in their medical history. Additionally, this approach can incorporate general knowledge and known risk factors into research pertaining to a given disease we are trying to predict.

## Conclusion

In this work, we presented CPLLM, a novel method for the prediction of clinical disease diagnoses and patient hospital readmission based on the medical history of a given patient. CPLLM has the potential for practical application. By surpassing the state-of-the-art in clinical task prediction performance, our method enables more accurate and robust disease forecasting, and can more reliably gauge the odds of patient hospital readmission. CPLLM demonstrated superior performance across all four tasks on two different datasets (MIMIC-IV and eICU-CRD). It processes ICD9 and ICD10 diagnoses, procedures, and drugs to inform its predictions. We showcased its robustness in dealing with homogeneous and multi-center data. Our method’s advantage lies in eliminating any need for additional pre-training tasks, in contrast with Med-BERT. Furthermore, our method remains adaptable even when information pertaining to length of stay data is unavailable, making it suitable for a broader range of healthcare scenarios, including those involving non-hospitalized patients. In addition, CPLLM’s fine-tuning process requires patients’ diagnoses as a sequence, without requiring information regarding which diagnoses were made during which visit. Notably, our method can handle much longer sequences than existing state-of-the-art models.

We believe that CPLLM has significant practical applications. For instance, healthcare stakeholders are increasingly seeking methods to enhance patient care without compromising data privacy. The two LLMs we tested can be deployed and utilized on-site or in secure environments, eliminating the need to share personal data over the internet.

## Supporting information

S1 Fig Precision-recall curves. Precision-Recall for predicting readmission in the MIMIC-IV dataset, showcasing the performance of the two best models, CPLLM-Llama2 and ConCare.
(EPS)

S2 Fig ROC curves. Receiver Operating Characteristic curves for predicting readmission in the MIMIC-IV dataset, showcasing the performance of the two best models, CPLLM-Llama2 and ConCare.
(EPS)

S3 Fig Precision-recall curves comparison: CPLLM-Llama2 vs. Med-BERT. Comparison of CPLLM-Llama2 and Med-BERT using Precision-Recall for predicting Adult Respiratory Failure in the eICU-CRD dataset.
(EPS)

S4 Fig ROC curves comparison: CPLLM-Llama2 vs. Med-BERT. Comparison of CPLLM-Llama2 and Med-BERT using ROC curves for predicting Adult Respiratory Failure in the eICU-CRD dataset.
(EPS)
